# Poly[3,3′-diethyl-1,1′-(ethane-1,2-di­yl)diimidazolium [tetra-μ-bromido-­diargentate(I)]]

**DOI:** 10.1107/S160053681002146X

**Published:** 2010-06-26

**Authors:** Zhiguo Wang, Siman Liu, Na Zhang

**Affiliations:** a Department of Chemistry and Chemical Engineering, Mianyang Normal University, Mianyang 621000, People’s Republic of China

## Abstract

The asymmetric unit of the title salt, {(C_12_H_20_N_4_)[Ag_2_Br_4_]}_*n*_, contains one-half of a substituted imidazolium cation, one Ag^+^ and two Br^−^ ions. The cation is completed by crystallographic inversion symmetry. The crystal structure is made up from polymeric sheets of {[AgBr_2_]^−^}_*n*_ anions extending parallel to (100). The basic building unit of the anion is a slightly distorted AgBr_4_ tetra­hedron. A four- and 12-membered ring system is formed by corner sharing of the AgBr_4_ tetra­hedra. The imidazolium cations are located between the anionic sheets and partly protrude into the voids defined by the 12-membered rings.

## Related literature

For general background to *N*-heterocyclic carbenes, see: Arnold (2002 ▶); Lin & Vasam (2004 ▶). For related structures, see: Lee *et al.* (2002 ▶); Helgesson & Jagner (1990 ▶, 1991 ▶); Olson *et al.* (1994 ▶).
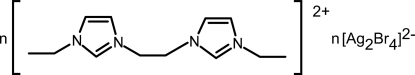


## Experimental

### 

#### Crystal data

(C_12_H_20_N_4_)[Ag_2_Br_4_]
*M* *_r_* = 755.90
Monoclinic, 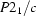

*a* = 9.5593 (13) Å
*b* = 12.9512 (17) Å
*c* = 8.4565 (11) Å
β = 106.294 (2)°
*V* = 1004.9 (2) Å^3^
*Z* = 2
Mo *K*α radiation
μ = 9.90 mm^−1^
*T* = 296 K
0.25 × 0.24 × 0.22 mm

#### Data collection

Bruker SMART CCD area-detector diffractometer
Absorption correction: multi-scan (*SADABS*; Sheldrick, 2006 ▶) *T*_min_ = 0.191, *T*_max_ = 0.219
5036 measured reflections
1766 independent reflections
1533 reflections with *I* > 2σ(*I*)
*R*_int_ = 0.023

#### Refinement

*R*[*F* ^2^ > 2σ(*F* ^2^)] = 0.031
*wR*(*F* ^2^) = 0.077
*S* = 1.06
1766 reflections
101 parameters
H-atom parameters constrained
Δρ_max_ = 0.64 e Å^−3^
Δρ_min_ = −0.88 e Å^−3^

### 

Data collection: *SMART* (Bruker, 2006 ▶); cell refinement: *SAINT* (Bruker, 2006 ▶); data reduction: *SAINT*; program(s) used to solve structure: *SHELXS97* (Sheldrick, 2008 ▶); program(s) used to refine structure: *SHELXL97* (Sheldrick, 2008 ▶); molecular graphics: *DIAMOND* (Crystal Impact, 2008 ▶); software used to prepare material for publication: *SHELXL97*.

## Supplementary Material

Crystal structure: contains datablocks I, global. DOI: 10.1107/S160053681002146X/wm2354sup1.cif

Structure factors: contains datablocks I. DOI: 10.1107/S160053681002146X/wm2354Isup2.hkl

Additional supplementary materials:  crystallographic information; 3D view; checkCIF report

## Figures and Tables

**Table:** 

Ag1—Br1	2.6788 (7)
Ag1—Br2^i^	2.6934 (8)
Ag1—Br1^ii^	2.6999 (7)
Ag1—Br2	2.7227 (8)

**Table:** 

Br1—Ag1—Br2^i^	114.34 (3)
Br1—Ag1—Br1^ii^	103.92 (2)
Br2^i^—Ag1—Br1^ii^	116.12 (3)
Br1—Ag1—Br2	119.16 (3)
Br2^i^—Ag1—Br2	95.81 (2)
Br1^ii^—Ag1—Br2	107.93 (2)

Symmetry codes: (i) 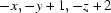; (ii) 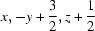.

**Fig. 1.**
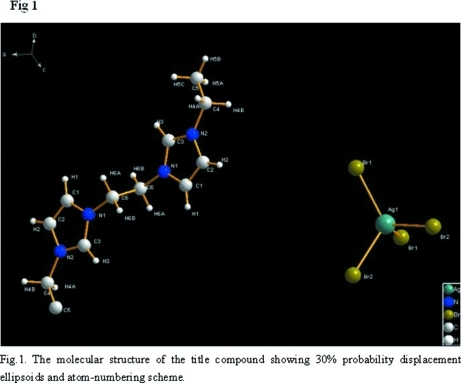
The [(C12H20N4)]2+ cation and the basic AgBr4 building unit of the polymeric anion. Displacement ellipsoids are drawn at the 30% probability level.

**Fig. 2.**
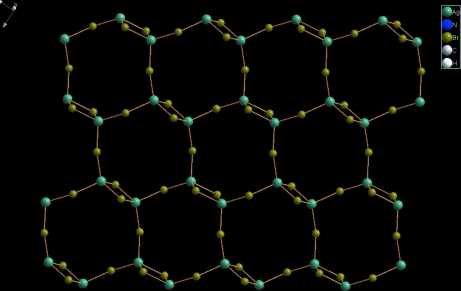
The four- and twelve-membered ring system of the polymeric {[Ag2Br4]2-}n anion.

## supplementary crystallographic information

### Comment

Silver and other transition metal N-heterocyclic carbene complexes have played an important role in the development of metal-carbene systems for transmetalation reactions. Silver oxide is the most commonly used metal base for this purposes. Recent reviews dealing with silver N-heterocyclic carbenes were published by Arnold (2002) and Lin & Vasam (2004). The products differ depending upon reaction conditions and the imidazolium salt used. The silver carbene [Ag_2_(Me_2_-edimy)Cl_2_] has been successfully synthesized by the reaction of [Me_2_-edimyH_2_][PF_6_]_2_ with Ag_2_O in CH_3_CN and [NM_4_]Cl (Lee *et al.*, 2002). In an attempt to prepare a similar carbene, we obtained the title compound, [(C_12_H_20_N_4_)]^2+^[Ag_2_Br_4_]^2-^, instead. Synthesis and crystal structure are reported in this article.

The crystal structure of the title salt is composed of [(C_12_H_20_N_4_)]^2+^ cations and [Ag_2_Br_4_]^2-^ anions (Fig. 1). The anion forms polymeric sheets extending parallel to (100). The cations are located between the sheets and partly reach through the voids of the anion. A characteristic feature of the polymeric {[Ag_2_Br_4_]^2-^}_n_ anion is the construction of rings built up from corner-sharing of slightly distorted AgBr_4_ tetrahedra. A large twelve-membered ring is formed by six alternating bromine and six silver atoms; another four-membered ring completes the building units of the polymeric anion (Fig. 2). The four-membered ring is very similar to that in the complex anion [Ag_4_Br_8_]^4-^ (Helgesson & Jagner, 1991). These anions contain tetrahedrally coordinated Ag^+^ atoms, whereas the [Ag_4_I_8_]^4-^ ion, isolated as the tetraphenylphosphonium and tetraphenylarsonium salts, contains three-coordinated and four-coordinated Ag^+^ (Helgesson & Jagner, 1990).

The average Ag—Br distance of the AgBr_4_ tetrahedron in the title compound is 2.699 Å, which is considerably longer than for the [Ag_2_Br_4_]^2-^ dimer ((2.518 (2) Å; Helgesson *et al.*, 1990). These values are comparable to other tetrahedral AgBr_4_ units (Olson *et al.*, 1994).

### Experimental

Ag_2_O (2.32 g, 10 mmol) was added to a solution of 1H-imidazolium, 1,1'-(1,2-ethanediyl)bis[3-ethyl] dibromide (3.78 g, 10 mmol) in DMSO. This mixture was refluxed for 30 min under stirring, resulting in a clean solution. When the solvent was removed, the residue was exatracted with acetonitrile. The remaining residue was separated by centrifugation and the resulting solution was kept at room temperature. Colourless crystals of the title compound were obtained after slow evaporation (2.64 g, 34.9 % yield). Mp: 421 K. ^1^H NMR (CDCl_3_): 9.48(m,1H), 9.43 (m.1H), 6.84 (s, 2H, CH), 6.87 (s, 2H, CH), 4.52 (s, 4H, CH2), 3.64(s, 4H, CH3),1.42(m, 6H) ppm. Anal. calcd.: C, 19.05 H, 2.65; N, 7.41; found: C, 19.26; H, 2.57 ; N, 7.32%.

### Refinement

The H atoms attached to C atoms of the imidazole ring were positioned geometrically and allowed to ride on their parent atoms, with a C—H distance of 0.93 Å and *U*_iso_(H) = 1.2*U*_eq_(C). Methylene and methyl H atoms were likewise positioned geometrically and refined as riding atoms, with C—H = 0.97 Å (methylene) and C—H = 0.96 Å (methyl) and *U*_iso_(H) = 1.2*U*_eq_(C).

### Crystal data

**Table:**

(C_12_H_20_N_4_)[Ag_2_Br_4_]	*F*(000) = 708
*M**_r_* = 755.90	*D*_x_ = 2.498 Mg m^−^^3^
Monoclinic, *P*2_1_/*c*	Mo *K*α radiation, λ = 0.71073 Å
Hall symbol: -P 2ybc	Cell parameters from 3112 reflections
*a* = 9.5593 (13) Å	θ = 2.2–27.8°
*b* = 12.9512 (17) Å	µ = 9.90 mm^−^^1^
*c* = 8.4565 (11) Å	*T* = 296 K
β = 106.294 (2)°	Block, colourless
*V* = 1004.9 (2) Å^3^	0.25 × 0.24 × 0.22 mm
*Z* = 2	

### Data collection

**Table:**

Bruker SMART CCD area-detector diffractometer	1766 independent reflections
Radiation source: fine-focus sealed tube	1533 reflections with *I* > 2σ(*I*)
graphite	*R*_int_ = 0.023
phi and ω scans	θ_max_ = 25.0°, θ_min_ = 2.7°
Absorption correction: multi-scan (*SADABS*; Sheldrick, 2006)	*h* = −11→11
*T*_min_ = 0.191, *T*_max_ = 0.219	*k* = −15→14
5036 measured reflections	*l* = −10→5

### Refinement

**Table:**

Refinement on *F*^2^	Primary atom site location: structure-invariant direct methods
Least-squares matrix: full	Secondary atom site location: difference Fourier map
*R*[*F*^2^ > 2σ(*F*^2^)] = 0.031	Hydrogen site location: inferred from neighbouring sites
*wR*(*F*^2^) = 0.077	H-atom parameters constrained
*S* = 1.06	*w* = 1/[σ^2^(*F*_o_^2^) + (0.0366*P*)^2^ + 1.7373*P*] where *P* = (*F*_o_^2^ + 2*F*_c_^2^)/3
1766 reflections	(Δ/σ)_max_ = 0.001
101 parameters	Δρ_max_ = 0.64 e Å^−^^3^
0 restraints	Δρ_min_ = −0.88 e Å^−^^3^

### Special details

**Table:**

Geometry. All esds (except the esd in the dihedral angle between two l.s. planes) are estimated using the full covariance matrix. The cell esds are taken into account individually in the estimation of esds in distances, angles and torsion angles; correlations between esds in cell parameters are only used when they are defined by crystal symmetry. An approximate (isotropic) treatment of cell esds is used for estimating esds involving l.s. planes.
Refinement. Refinement of *F*^2^ against ALL reflections. The weighted *R*-factor wR and goodness of fit *S* are based on *F*^2^, conventional *R*-factors *R* are based on *F*, with *F* set to zero for negative *F*^2^. The threshold expression of *F*^2^ > σ(*F*^2^) is used only for calculating *R*-factors(gt) etc. and is not relevant to the choice of reflections for refinement. *R*-factors based on *F*^2^ are statistically about twice as large as those based on *F*, and *R*- factors based on ALL data will be even larger.

### Fractional atomic coordinates and isotropic or equivalent isotropic displacement parameters (Å^2^)

**Table:**

	*x*	*y*	*z*	*U*_iso_*/*U*_eq_	
Ag1	0.06806 (5)	0.63160 (3)	1.01898 (5)	0.05733 (16)	
N1	0.8101 (4)	0.5496 (3)	0.4321 (5)	0.0424 (9)	
N2	0.6179 (5)	0.6042 (4)	0.2519 (6)	0.0575 (12)	
Br1	0.13558 (8)	0.75994 (4)	0.80302 (6)	0.0632 (2)	
Br2	0.20441 (6)	0.44528 (4)	1.08097 (7)	0.05774 (18)	
C1	0.6983 (6)	0.4833 (5)	0.4277 (8)	0.0646 (16)	
H1	0.7040	0.4239	0.4911	0.078*	
C2	0.5809 (6)	0.5178 (5)	0.3180 (8)	0.0650 (16)	
H2	0.4889	0.4877	0.2912	0.078*	
C3	0.7579 (6)	0.6221 (5)	0.3208 (7)	0.0584 (14)	
H3	0.8110	0.6766	0.2952	0.070*	
C4	0.5147 (8)	0.6630 (6)	0.1169 (10)	0.095 (3)	
H4A	0.4988	0.6241	0.0153	0.114*	
H4B	0.4219	0.6683	0.1417	0.114*	
C5	0.5611 (12)	0.7605 (6)	0.0923 (13)	0.121 (4)	
H5A	0.5711	0.8012	0.1898	0.182*	
H5B	0.4911	0.7923	0.0012	0.182*	
H5C	0.6535	0.7563	0.0688	0.182*	
C6	0.9600 (5)	0.5390 (4)	0.5370 (6)	0.0432 (11)	
H6A	0.9594	0.5169	0.6464	0.052*	
H6B	1.0093	0.6052	0.5469	0.052*	

### Atomic displacement parameters (Å^2^)

**Table:**

	*U*^11^	*U*^22^	*U*^33^	*U*^12^	*U*^13^	*U*^23^
Ag1	0.0590 (3)	0.0526 (3)	0.0561 (3)	−0.0056 (2)	0.0091 (2)	0.00076 (18)
N1	0.031 (2)	0.048 (2)	0.045 (2)	−0.0008 (18)	0.0046 (17)	0.0031 (18)
N2	0.039 (3)	0.074 (3)	0.053 (3)	0.005 (2)	0.002 (2)	0.013 (2)
Br1	0.1030 (5)	0.0489 (3)	0.0362 (3)	−0.0160 (3)	0.0171 (3)	0.0008 (2)
Br2	0.0326 (3)	0.0547 (3)	0.0783 (4)	0.0051 (2)	0.0029 (2)	−0.0090 (3)
C1	0.039 (3)	0.058 (3)	0.088 (4)	−0.008 (3)	0.002 (3)	0.023 (3)
C2	0.035 (3)	0.068 (4)	0.083 (4)	−0.006 (3)	0.002 (3)	0.014 (3)
C3	0.045 (3)	0.068 (4)	0.061 (4)	−0.005 (3)	0.013 (3)	0.017 (3)
C4	0.064 (5)	0.106 (6)	0.096 (6)	0.003 (4)	−0.007 (4)	0.043 (5)
C5	0.135 (9)	0.067 (5)	0.125 (8)	0.012 (5)	−0.026 (6)	0.003 (5)
C6	0.035 (3)	0.051 (3)	0.040 (3)	−0.004 (2)	0.005 (2)	−0.008 (2)

### Geometric parameters (Å, °)

**Table:**

Ag1—Br1	2.6788 (7)	C2—H2	0.9300
Ag1—Br2^i^	2.6934 (8)	C3—H3	0.9300
Ag1—Br1^ii^	2.6999 (7)	C4—C5	1.374 (10)
Ag1—Br2	2.7227 (8)	C4—H4A	0.9700
N1—C3	1.324 (6)	C4—H4B	0.9700
N1—C1	1.363 (7)	C5—H5A	0.9600
N1—C6	1.466 (6)	C5—H5B	0.9600
N2—C3	1.321 (7)	C5—H5C	0.9600
N2—C2	1.341 (7)	C6—C6^iii^	1.506 (9)
N2—C4	1.491 (8)	C6—H6A	0.9700
C1—C2	1.317 (8)	C6—H6B	0.9700
C1—H1	0.9300		
			
Br1—Ag1—Br2^i^	114.34 (3)	N2—C3—H3	125.6
Br1—Ag1—Br1^ii^	103.92 (2)	N1—C3—H3	125.6
Br2^i^—Ag1—Br1^ii^	116.12 (3)	C5—C4—N2	114.4 (7)
Br1—Ag1—Br2	119.16 (3)	C5—C4—H4A	108.7
Br2^i^—Ag1—Br2	95.81 (2)	N2—C4—H4A	108.7
Br1^ii^—Ag1—Br2	107.93 (2)	C5—C4—H4B	108.7
C3—N1—C1	106.9 (4)	N2—C4—H4B	108.7
C3—N1—C6	127.3 (4)	H4A—C4—H4B	107.6
C1—N1—C6	125.7 (4)	C4—C5—H5A	109.5
C3—N2—C2	108.4 (5)	C4—C5—H5B	109.5
C3—N2—C4	128.2 (5)	H5A—C5—H5B	109.5
C2—N2—C4	123.4 (5)	C4—C5—H5C	109.5
Ag1—Br1—Ag1^iv^	152.39 (4)	H5A—C5—H5C	109.5
Ag1^i^—Br2—Ag1	84.19 (2)	H5B—C5—H5C	109.5
C2—C1—N1	108.1 (5)	N1—C6—C6^iii^	109.6 (5)
C2—C1—H1	125.9	N1—C6—H6A	109.8
N1—C1—H1	125.9	C6^iii^—C6—H6A	109.8
C1—C2—N2	107.8 (5)	N1—C6—H6B	109.8
C1—C2—H2	126.1	C6^iii^—C6—H6B	109.8
N2—C2—H2	126.1	H6A—C6—H6B	108.2
N2—C3—N1	108.8 (5)		
			
Br2^i^—Ag1—Br1—Ag1^iv^	4.51 (7)	C4—N2—C2—C1	177.1 (7)
Br1^ii^—Ag1—Br1—Ag1^iv^	−123.08 (5)	C2—N2—C3—N1	−1.2 (7)
Br2—Ag1—Br1—Ag1^iv^	116.83 (6)	C4—N2—C3—N1	−177.9 (7)
Br1—Ag1—Br2—Ag1^i^	−122.09 (3)	C1—N1—C3—N2	1.7 (7)
Br2^i^—Ag1—Br2—Ag1^i^	0.0	C6—N1—C3—N2	179.7 (5)
Br1^ii^—Ag1—Br2—Ag1^i^	119.87 (3)	C3—N2—C4—C5	−18.3 (12)
C3—N1—C1—C2	−1.5 (7)	C2—N2—C4—C5	165.6 (8)
C6—N1—C1—C2	−179.6 (5)	C3—N1—C6—C6^iii^	−98.5 (7)
N1—C1—C2—N2	0.8 (8)	C1—N1—C6—C6^iii^	79.1 (7)
C3—N2—C2—C1	0.2 (8)		

Symmetry codes: (i) −*x*, −*y*+1, −*z*+2; (ii) *x*, −*y*+3/2, *z*+1/2; (iii) −*x*+2, −*y*+1, −*z*+1; (iv) *x*, −*y*+3/2, *z*−1/2.
